# The effect of urine storage temperature and boric acid preservation on quantitative bacterial culture for diagnosing canine urinary tract infection

**DOI:** 10.1186/s12917-021-03083-6

**Published:** 2021-12-08

**Authors:** M. Hedström, M. Møller, H. Patsekhina, P. Damborg, L. R. Jessen, T. M. Sørensen

**Affiliations:** 1 Department of Veterinary Clinical Sciences, Faculty of Health and Medical Sciences, University of Copenhagen, Dyrlaegevej 16, 1870 Frederiksberg C, Denmark; 2 Department of Veterinary and Animal Sciences, Faculty of Health and Medical Sciences, University of Copenhagen, Stigbøjlen 4, 1870 Frederiksberg C, Denmark

**Keywords:** Dog, Urine preservation, Urine transportation, Urine cultures, Refrigeration, Significant bacteriuria

## Abstract

**Background:**

Quantitative bacterial culture (QBC) is the gold standard for diagnosing canine urinary tract infection. Current guidelines recommend QBC within 24 h of urine collection and that unpreserved urine is refrigerated until culture. However, temperature-controlled transport is rarely feasible, indicating a need for alternative storage during transport of urine from primary veterinary practices to the microbiology laboratory.

The objective was to investigate the effect of storage temperature and boric acid sponge-preservation on quantitative bacterial culture of canine urine.

**Results:**

Significant bacteriuria was detected in 72 out of 179 samples (40%) collected from 141 dogs. Overall accuracy was 94–98% for both storage conditions and time points. Non-inferiority (15% margin) to reference quantitative bacterial culture was evident for sensitivity, specificity and predictive values for both storage methods and time points, except for the negative predictive value for 48 h boric acid preservation (NPV: 89, 95% CI [79;95]). There was no significant difference between the sensitivity and specificity for either of the time-points (*p*-value = 0.07–1).

**Conclusions:**

Boric acid sponge-preservation using Uriswab™ is a useful alternative to refrigeration of urine samples during transport. Reliable quantitative bacterial culture results can be obtained from canine urine up to 48 h after collection if urine is refrigerated, and for at least 24 h if urine is stored using a boric acid-containing urine transport system.

## Background

Dogs presenting with clinical signs of lower urinary tract disease (LUTD) are commonly encountered in companion animal practice. Approximately 38–65% of dogs with signs of LUTD have a bacterial cystitis [1–3] making diagnostic work-up essential for identifying cases with true infection.

Quantitative bacterial culture (QBC) with significant bacteriuria and compatible clinical signs is the gold standard for diagnosing urinary tract infection (UTI). Current international guidelines recommend QBC within 24 h of urine collection and that urine, if unpreserved, is stored and transported refrigerated to maintain pathogen viability and quantity [4]. However, temperature-controlled transport can be more expensive and may not always be feasible, indicating a need for alternative storage methods during transport.

Urine transport systems containing boric acid (BA) preservative are commonly recommended and described in human medicine [5–9], but research of their application in companion animal medicine is limited.

Stable bacterial counts have been identified in canine urine collected by various methods when preserved for up to 72 h in commercial products containing BA [10, 11]. However, excess inhibition of bacterial growth has been demonstrated in canine urine preserved with very high concentrations (10 to 20 g/L) of BA [12], highlighting the importance of a correct BA/urine ratio. Available commercial BA tubes are designed for use in humans and require 3–10 mL of urine to ensure a correct BA to urine ratio. Such a volume can be challenging to obtain in some companion animals with clinical signs of UTI.

The aim of this study was to investigate the effect of storage temperature and BA preservation on QBC of canine urine, using a commercial system composed of a urine preservation sponge with volume-dependent BA release (UriSwab™). The principle of this preservation method is for BA to be released only in the area of the sponge embedded in urine, hereby ensuring an adequate BA/urine ratio irrespective of urine volume. We hypothesized that i) QBC on non-preserved refrigerated urine or on urine preserved in the above-mentioned BA commercial system at room-temperature, for 24 and 48 h respectively, is non-inferior to the QBC performed immediately upon urine collection; ii) there is no significant difference in sensitivity and specificity of the two storage methods.

## Results

**Fig. 1:**
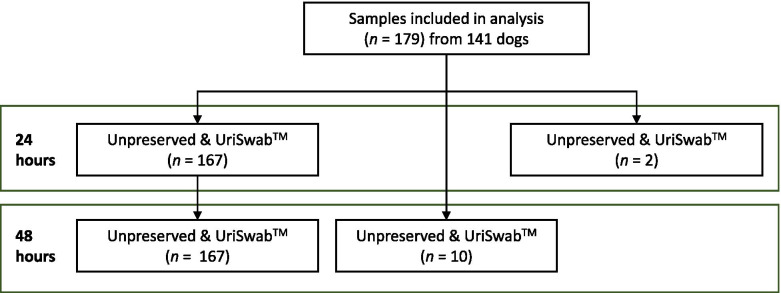
Flow diagram of urine samples enrolled and included in analysis after 24 and 48 h, respectively. Out of 170 samples included, 10 samples were prioritized for 48 h because of insufficient volume and two samples were not cultured at 48 h due to lack of access to the laboratory on the specific days needed. Unpreserved samples were stored refrigerated while boric acid preserved (UriSwab^TM^) samples were stored at room temperature

One hundred and seventy-nine urine samples from 141 dogs (77 (43%) male, 87 (49%) female and 15 (8%) without a recorded sex) were included in the study. The median age of the study population was 7 years (range 0.25–14 years). Ten samples were cultured only after 48 h of storage because of insufficient volume for both time points (Fig. 1). Two samples were cultured after 24 but not 48 h, as the latter time point fell outside laboratory opening hours.

The majority of samples, 99 (55%) were collected by cystocentesis, while voided midstream accounted for 52 (29%), and catheterization for 18 (10%). For the remaining 19 (6%) samples, the collection method was not provided on the laboratory submission form or available from the medical records, and therefore classified as unknown.

At time zero, reference QBC showed significant bacteriuria in 72 (40%) of the samples. The most commonly recovered organism were, *Escherichia coli* (*n* = 36), *Proteus mirabilis* (*n* = 11), *Staphylococcus* spp. (*n* = 7) and *Streptococcus* spp. (*n* = 4). Two samples yielded mixed cultures and from two samples cultured bacteria could not be identified to the species level using MALDI-TOF.

After 24 h of storage, significant bacteriuria was observed in 69 of 169 samples (41%) with COOL storage, and 65 (38%) for ROOM storage. After 48 h of storage, significant bacteriuria was observed in 72 of the 177 samples (41%) with COOL storage, and 66 (37%) for ROOM storage. An overall accuracy of 94–98% was found for all storage conditions (Table 1).

**Table 1:** Calculated accuracy, sensitivity, specificity, positive and negative predictive value for significant bacteriuria compared to reference quantitative bacterial culture. Brachets is one-sided 95% confidence interval limits

Storage condition					Predictive value		
Time	Method	*n*	Sensitivity	Specificity	Positive	Negative	Accuracy
24 h	Non-preserved, COOL	169	0.98	0.94	0.96	0.97	97%
			(0.93–1.00)	(0.86–0.98)	(0.90–0.99)	(0.90–1.00)	
	UriSwab™, ROOM	169	0.97	0.98	0.99	0.96	98%
			(0.91–0.99)	(0.92–1.00)	(0.95–1.00)	(0.87–0.99)	
48 h	Non-preserved, COOL	177	0.97	0.95	0.97	0.96	96%
			(0.92–0.99)	(0.88–0.99)	(0.92–0.99)	(0.88–0.99)	
	UriSwab™, ROOM	177	0.93	0.97	0.98	0.89	94%
			(0.86–0.97)	(0.89–1.00)	(0.93–1.00)	(0.79–0.95)	

*COOL* Urine stored refrigerated, *ROOM* Urine stored in boric acid (UriSwab™) at room temperature

Calculated accuracy, sensitivity, specificity, positive and negative predictive value for significant bacteriuria compared to reference quantitative bacterial culture. Brackets is one-sided 95% confidence interval limits

**Fig. 2:**
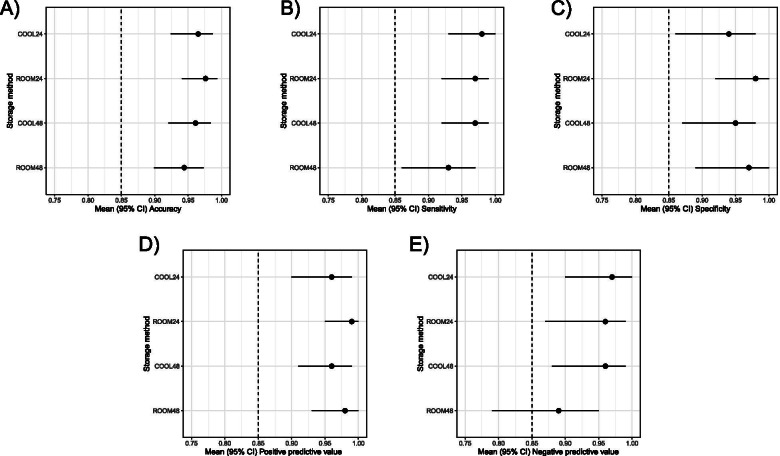
The results of non-inferiority trials relating to accuracy, sensitivity, specificity, positive and negative predictive value for COOL and ROOM at 24 and 48 h, respectively, with a non-inferiority margin set at 15% (Δ = 0.15). A-D) The lower bound of the 95% confidence interval (CI) does not fall below the noninferiority margin and non-inferiority is demonstrated for all storage methods and timepoints. E) The lower bound of the 95% CI falls below the non-inferiority margin (0.85) for the negative predictive value at 48 h and non-inferiority could thereby not be demonstrated. Remaining storage methods and timepoints demonstrates non-inferiority. COOL: Urine stored refrigerated; ROOM: Urine stored in boric acid (UriSwab™) at room temperature. Number indicates time interval since reference culture

Non-inferiority to reference QBC was evident for all of the storage methods, except for ROOM at 48 h where non-inferiority could not be proven for the negative predictive value (89, 95% CI [79 to 95]) (Fig. 2).

When comparing COOL and ROOM storage methods using McNemar’s χ^2^-test, there was no significant difference between the sensitivity and specificity for either of the time points. At 24 h, the sensitivity for COOL was 98% and ROOM 97% (*p*-value = 1), and the specificity for COOL 94% and ROOM 98% (*p*-value = 0.37). At 48 h, the sensitivity for COOL was 97% and ROOM 93% (*p*-value = 0.07), and the specificity for COOL 96% and ROOM 97% (*p*-value = 1).

A variation in CFU/ml level was observed for one or more storage conditions in 83 of 179 samples, though only influencing the conclusion regarding significant bacteriuria in 16 samples (Table 2). False positive results were obtained at one or both time points compared to reference in COOL (*n* = 7) and ROOM (*n* = 3) samples, and false negative results in COOL (*n* = 3) and ROOM (*n* = 11) samples.

**Table 2:** Colony forming units (CFU)/mL rounded down to the nearest whole logarithmic number for 16 samples with discrepant counts resulting in altered conclusion regarding significant bacteriuria

	Reference		COOL 24 h		COOL 48 h		ROOM 24 h		ROOM 48 h	
Collection method	CFU/mL	UTI	CFU/mL	UTI	CFU/mL	UTI	CFU/mL	UTI	CFU/mL	UTI
Cystocentesis	Sterile	No	Sterile	No	10^5^	Yes^a^	Sterile	No	Sterile	No
	Sterile	No	10^3^	Yes^a^	Sterile	No	Sterile	No	10^5^	No
	Sterile	No	10^3^	Yes^a^	10^5^	No	10^2^	No	Sterile	No
	10^2^	No	Sterile	No	10^3^	Yes^a^	Sterile	No	Sterile	No
	10^4^	Yes	10^4^	Yes	10^5^	Yes	10^4^	Yes	10^2^	No^b^
	10^5^	Yes	10^4^	Yes	10^3^	Yes	10^3^	Yes	10	No^b^
	10^5^	Yes	10^5^	Yes	10^2^	No^b^	10^5^	Yes	Sterile	No^b^
Catheter	Sterile	No	Sterile	No	10^4^	No	10^5^	Yes^a^	10^5^	Yes^a^
Voided	10^4^	No	10^5^	Yes^a^	10^5^	Yes^a^	10^4^	No	10^5^	Yes^a^
	10^4^	No	10^5^	Yes^a^	10^4^	No	10^4^	No	10^4^	No
	10^5^	Yes	10^4^	No^b^	10^5^	Yes	10^4^	No^b^	10^4^	No^b^
	10^5^	Yes	10^5^	Yes	10^4^	No^b^	10^4^	No^b^	10^3^	No^b^
	10^5^	Yes	10^4^	No^b^	10^5^	Yes	10^5^	Yes	10^5^	Yes
	10^5^	Yes	10^5^	Yes	10^4^	No^b^	10^5^	Yes	10^4^	No^b^
	10^5^	Yes	10^5^	Yes	10^5^	Yes	10^4^	No^b^	10^4^	No^b^
	10^5^	Yes	10^5^	Yes	10^5^	Yes	10^5^	Yes	10^4^	No^b^

^a^False positive results compared to reference, ^b^False negative results compared to reference

*COOL* Urine stored refrigerated, *ROOM* Urine stored in boric acid (UriSwab™) at room temperature

Colony forming units (CFU)/mL rounded down to the nearest whole logarithmic number for 16 samples with discrepant counts resulting in altered conclusion regarding significant bacteriuria

## Discussion

The purpose of this study was to investigate the effect of storage temperature and BA-sponge preservation on QBC of canine urine collected by various methods for up to 48 h. Good agreement was found between reference urine culture and urine culture results obtained after 24 h of storage for both refrigerated urine (98% sensitivity, 94% specificity) and urine preserved in BA sponges (UriSwab™) at room temperature (97% sensitivity, 98% specificity) (Table 1). These results are comparable to previous reports on BA preservation of canine urine [10, 11, 13], and suggest that storage in BA is indeed a useful alternative to refrigeration of urine during transportation also in veterinary practice.

Good agreement was also found after 48 h of refrigeration (98% specificity, 95% sensitivity). However, for samples preserved with BA at room temperature, the sensitivity dropped at 48 h (from 97% to 93%) and non-inferiority could not be demonstrated for the calculated negative predictive value (89%, 95% CI [79% to 95%]) (Table 1). This could potentially lead to misdiagnosis and under-treatment of the individual patient and secondarily pose a risk for complications. However, it should be noted that the non-inferior margin is still included within the 95% CI, and it cannot be ruled out that the performance of ROOM at 48 h would be non-inferior with more samples included. Importantly, both storage methods at both time-points, including BA preservation at 48 h, performed markedly better compared to what has been shown for unpreserved urine sent by mail delivery or stored at room temperature, where up to 50% false positive growth and 4% false negative growth was observed at 24 h time points [13].

Boric acid preservation of human urine samples have been shown comparable to 24 h of refrigeration [14], and several countries and microbiology laboratories recommend the use of boric acid preservation for transportation of human urine from primary care facilities. Daley et al., (2018) reviewed the Copan UriSwab™ collection system in human urine and found that refrigeration without preservation and preservation with UriSwab™ had similar properties after 24, 48 and 72 h of storage. However, they noted an increase in significant bacteriuria for both storage methods compared to time zero reference culture after 24 h of storage, which then further increased up to 72 h. Our study demonstrated similar results to human studies comparing preservation with UriSwab™ versus BD Vacutainer C&S Preservative Plus (2.63 mg/mL boric acid, 3.95 mg/mL sodium borate and 1.65 mg/mL sodium formate) for 24 h [15, 16], and 48 h [17], where they found the two systems to have comparable results for maintaining viability of uropathogens. In addition, Rennie et al., (2016), found fewer mixed cultures in the samples collected by UriSwab™ versus BD Vacutainer C&S Preservative Plus and when comparing UriSwab™ to culture paddle the UriSwab™ was found to be superior to culture paddle in terms of maintaining uropathogens. However, none of these studies used a refrigerated control.

There are two recent studies on the use of BA for preservation of veterinary urine specimens [12, 18]. Rowlands et al. (2011) collected canine and feline urine by cystocentesis and stored urine in containers with a final BA concentration of 10 to 20 g/L. They argued against preservation with BA for transportation due to a high level of false negative results. However, the high level of false negative samples in that study (27%) is likely due to a mismatch of the urine/BA ratio leading to bactericidal concentrations of BA. In comparison, the UriSwab™ contains 6.5 mg/mL boric acid and 3.75 mg/mL sodium formate while the BD Vacutainer® Plus C&S Boric Acid Sodium Borate/Formate Tube contains 2.63 mg/mL boric acid, 3.95 mg/mL sodium borate and 1.65 mg/mL sodium formate.

Patterson et al. (2016) pooled urine from four dogs and experimentally spiked the sterilized urine with independent cultures of *E. coli* before storage in a plain sterile tube and BA tubes (BD Vacutainer® Plus C&S Boric Acid Sodium Borate/Formate Tube) at 4 °C and 25 °C followed by QBC at 0, 8 and 24 h, respectively. They found a substantial decrease (38.1% versus 67.8% at 4 °C, and 99.7% versus 83.8% at 25 °C) in bacterial concentration from reference over a 24 h period, indicating a failing experimental model. That study had several limitations. Only one bacterial species was evaluated and there was a small number of samples. The urine was collected from animals without a bacterial cystitis and did thereby not contain inflammatory mediators that might have an impact on bacterial survival and growth [18]. The urine was also filter sterilized by first removing gross debris through a filter paper and then using a 0.2-μm filter sterilization device before spiking the urine, which can have affected bacterial survival. Also, as in the study by Rowland, preservative/urine ratio was higher than recommended by the manufacturer due to a limited volume of urine (1 mL versus recommended 4 mL urine in the tube) which likely impacted bacterial viability in preserved samples.

The studies by Patterson and Rowlands both demonstrate the importance of a proper preservative/urine ratio if BA preservation is to be successful. The sponge system evaluated in our study does not require a fixed volume of urine to obtain a correct ratio and in that respect, offers an advantage over traditional BA tubes when only small amount of urine can be obtained from the patient. Furthermore the UriSwab™ only marginally increase the initial cost compared to collection in a plain collection tube and is thus feasible for use in primary practice. It therefore offers a convenient and cost-effective method for storage and transportation compared to refrigerated temperature-controlled transport to the microbiology laboratory.

The present study included multiple samples from several dogs, thereby potentially causing a statistical bias. However, statistical simulations including only one sample per dog, changed the mean sensitivity and specificity with a maximum of only 0.01 (data not shown). This indicates a minimal impact of such bias on results and conclusions.

Our study included urine that was up to 7 days old and from animals either with or without clinical signs. Although this limits the possibility of assessing prevalence of significant bacteriuria and relative occurrence of bacteria, it would not affect the evaluation of the tested storage conditions, as QBC was conducted at time 0 on the same urine.

## Conclusion

This study demonstrates the usefulness of BA sponge preservation as an alternative to refrigeration of urine samples during transport. Reliable QBC results can be obtained from canine urine up to 48 h after collection if urine is refrigerated or stored at room temperature using Copan UriSwab™.

## Materials and methods

### Study design

The study was designed as a prospective, non-blinded, non-inferiority cohort study. The study was approved by the Ethical Administrative Committee at University Hospital for.

Companion Animals (journal number 2013–3). The study is reported in accordance with ARRIVE guidelines (https://arriveguidelines.org).

### Samples

Convenient inclusion of canine urine samples obtained for diagnostic purposes and received between February 2015 and March 2016 at the veterinary microbiology laboratory Sund Vet Diagnostik, Department of Veterinary and Animal Sciences, University of Copenhagen, were prospectively included in the study. Samples were included both from dogs with and without clinical signs of LUTD if urine were collected within the previous 7 days. Multiple, individual urine samples collected from the same animal were treated as unique samples. Urine samples were collected by cystocentesis, catheterization or voided midstream. If collection method was not registered on the laboratory submission form or the medical record, the collection method was registered as unknown.

### Reference quantitative bacterial culture

Upon arrival at the reference laboratory, reference QBC was performed by inoculating 1 μL and 10 μL of urine on separate halves of a 5% calf blood agar plate, respectively. This first QBC would be designated time zero (T0). After overnight incubation at 37 °C, the number of colony-forming units per ml (CFU/ml) urine was calculated as a weighted mean of colony counts determined from reading both halves of the agar [19]. For cultures with ≥10^3^ CFU/mL, each colony type was sub-cultured, and identified to the species level by matrix-assisted laser desorption/ionization time of flight (MALDI-TOF) mass spectrometry (Vitek MS RUO, bioMérieux; France) using *Escherichia coli* ATCC 8739 as reference strain and the software Saramis TM 3.5 (bioMérieux) for spectra interpretation. Significant bacteriuria was defined according to current recommendations as shown in Table 3. A blood agar plate was considered to be contaminated if there was considerable bacterial growth only on the half of the plate inoculated with 1 μL urine, or if bacterial growth was observed outside of the area streaked with urine.

**Table 3:** Criteria for classification as significant bacteriuria with regards to urine collection method

Veterinary ISCAID guidelines for significant bacteriuria^a^		
Collection method	Criteria (CFU/mL)	
Cystocentesis	≥ 1.000	
Catherization	≥ 100.000	
Voided	≥ 100.000	

^a^Weese et al., 2019 [4]. *ISCAID* International Society for Companion Animal Infectious Diseases

### BA preservation system

Copan UriSwab™ (Copan Italia, Brescia, Italy) is a urine preservation device containing boric acid (6.5 mg/ml) and sodium formate (3.75 mg/ml) preservatives chemically bonded into a sponge in a plastic tube. Preservatives are activated upon contact with urine, and the *stoichiometric* principle guarantees a mixture of urine and preservatives in correct proportions. The UriSwab™ sponge can hold a maximum of 1.5 mL of urine. The sponge was used according to the manufacturer’s instructions with few exceptions. In short, one sponge per study time point (T24 and T48) was dipped directly into a urine sample. If urine quantity was insufficient for dipping, all urine available was applied onto the sponge for absorption using a pipette. After storage at room temperature, the sponge was centrifuged at 500 RPM for 1 min to release urine from the sponge before QBC as described below.

### Culture upon storage of urine for 24 h and 48 h

Immediately after initial reference QBC, the remainder of a urine sample was split into four aliquots: i) unpreserved urine (50 μL) was stored refrigerated (4 °C) in a microfuge tube for 24 h (COOL, 24), ii) unpreserved urine (50 μL) was stored refrigerated (4 °C) in a microfuge tube for 48 h (COOL, 48), iii) BA preserved urine (0.1–1.5 mL) was stored at room temperature in a UriSwab™ system for 24 h (ROOM, 24), and iv) BA preserved urine (0.1–1.5 mL) was stored at room temperature in a UriSwab™ system for 48 h (ROOM, 48). If the urine volume was insufficient for all aliquots, priority was given to the 48 h time point.

QBC on all stored samples, at their respective time points, was performed and classified as described in section 5.3 for the reference QBC. All plates with bacterial growth were photographed and registered for future reference if any inconsistency were found in the results.

### Statistical analysis

All statistical analyses were performed using RStudio Version 1.4.1133 (RStudio Inc., Boston, MA, USA). Comparison between the two storage methods, the two time points, and the reference cultures for identifying significant bacteriuria (Table 3) were made by computing accuracy, sensitivity, specificity, positive, negative predictive value (PPV and NPV, respectively) and one-sided 95% confidence intervals using Confusion matrix and Epi-tests. The overall accuracy was defined as the ratio of (true positives (TP) + true negatives (TN)) divided by total number of cases. PPV is the proportion of positive QBC results that are true positives (the correct diagnosis according to the T0 reference QBC). NPV is the proportion of negative QBC results that are truly negative (according to the T0 reference QBC). Non-inferiority was concluded if the lower limit of the one-sided 95% confidence interval was above 0.85 (Δ = 0.15). McNemar’s Chi ^2^-test was used to compare sensitivity and specificity for the two storage methods.

## Data Availability

The dataset analyzed in this study is available from the UCPH ERDA data repository, and can be accessed through https://sid.erda.dk/share_redirect/hc3wF7yJIw.

## Abbreviations

QBC: Quantitative bacterial culture
BA: Boric acid
UTI: Urinary tract infection
ROOM: Storage boric acid (Copan Uriswab™) at room-temperature
COOL: Storage non-preserved by refrigeration
CFU: Colony-forming units
LUTD: Lower urinary tract disease
